# Neurocognitive impairment among HIV-positive individuals in Botswana: a pilot study

**DOI:** 10.1186/1758-2652-13-15

**Published:** 2010-04-20

**Authors:** Kathy Lawler, Mosepele Mosepele, Sarah Ratcliffe, Esther Seloilwe, Katherine Steele, Rudo Nthobatsang, Andrew Steenhoff

**Affiliations:** 1 Centre for the Study of HIV and AIDS, University of Botswana, Gaborone, Botswana; 2 Infectious Disease Care Center, Princess Marina Hospital, Gaborone, Botswana; 3 Center for AIDS Research, University of Pennsylvania, Philadelphia, PA, USA; 4 Department of Neurology, University of Pennsylvania, Philadelphia, PA, USA; 5 Department of Biostatistics & Epidemiology, University of Pennsylvania, Philadelphia, PA, USA; 6 Children's Hospital of Philadelphia, Philadelphia, PA, USA

## Abstract

**Background:**

The primary objective of this study was to determine the prevalence of neurocognitive impairment among HIV-positive individuals in Botswana, using the International HIV Dementia Scale (IHDS). We also compared performance on the IHDS with performance on tests of verbal learning/memory and processing speed, and investigated the association between performance on the IHDS and such variables as depression, age, level of education and CD4 count.

**Methods:**

We conducted a cross-sectional study of 120 HIV-positive individuals randomly selected from an outpatient HIV clinic in Gaborone, Botswana. Patients provided a detailed clinical history and underwent neuropsychological testing; measures of depression, daily activities and subjective cognitive complaints were recorded.

**Results:**

Despite the fact that 97.5% of subjects were receiving highly active antiretroviral therapy (HAART), 38% met criteria for dementia on the IHDS, and 24% were diagnosed with major depressive disorder. There was a significant association between neurocognitive impairment as measured by the IHDS and performance on the other two cognitive measures of verbal learning/memory and processing speed. Level of education significantly affected performance on all three cognitive measures, and age affected processing speed and performance on the IHDS. Depression and current CD4 count did not affect performance on any of the cognitive measures.

**Conclusions:**

The prevalence of neurocognitive impairment in HIV-positive individuals in Botswana is higher than expected, especially since almost all of the subjects in this study were prescribed HAART. This suggests the need to reconsider the timing of introduction of antiretroviral therapy in developing countries where HAART is generally not administered until the CD4 cell count has dropped to 200/mm^3 ^or below. The contribution of other factors should also be considered, such as poor central nervous system penetration of some antiretrovirals, drug resistance, potential neurotoxicity, and co-morbidities. Memory impairment and poor judgment may be underlying causes for behaviours that contribute to the spread of HIV and to poor adherence. It is important to identify these neurobehavioural complications of HIV so that effective treatments can be developed.

## Background

Individuals with HIV infection often experience neurological complications, including cognitive deficits, referred to as HIV-associated neurocognitive disorders (HAND). The specific pattern of neuropsychological deficits is attributed to damage to the fronto-striatal circuitry, which is commonly observed in HIV infection [1-3].

In recent years, HIV-related neurological disease has been increasingly recognized in resource-limited settings. One of the first multi-centre international studies was carried out with HIV-positive symptomatic and asymptomatic patients and HIV-negative subjects by the World Health Organization (WHO) in five countries: Thailand, Zaire, Kenya, Brazil, and Germany. Researchers found that neuropsychological impairment of symptomatic HIV-positive individuals ranged from 13% in Brazil to 19% in Zaire [4].

Since this study, there has been growing concern about the impact of HIV subtypes on disease progression. In Uganda, where clades A and D are the predominant subtypes, 31% of HIV-positive subjects met criteria for dementia, 47% mild cognitive impairment, and 22% no impairment on neuropsychological testing. The authors concluded that HIV dementia in the Uganda sample might be similar to the frequency of HIV dementia in the US in the pre-highly active antiretroviral therapy (HAART) era [5]. A follow-up study in Uganda of the risk factors for developing HAND demonstrated that advanced age and low CD4+ T cell count were the main risk factors for dementia [6].

However, some researchers have suggested that the incidence of HIV dementia may vary due to different viral clades [7]. For example, a study in Ethiopia evaluated the neurological complications of HIV (clade C) in HAART-naïve patients compared with matched HIV-negative controls and did not find impairment on the International HIV Dementia Scale (IHDS), which led the authors to hypothesize that HIV-related dementia may vary due to different viral clades [8]. In contrast, the Australia-Pacific NeuroAIDS Consortium found substantial neurocognitive and motor impairment in clade C HIV-positive subjects in several countries in the Pacific Rim. Timed gait was impaired in 28%, with impairment for verbal fluency in 33.6%, grooved peg board in 14%, and finger tapping in 43% [9].

Similarly, a study of HIV-positive subjects in southern India, where clade C is most common, found 60.5% had mild to moderate cognitive deficits in the domains of verbal fluency, working memory, and learning/memory in subjects who did not have clinically identifiable functional impairment [10]. Most recently, a study in China found neuropsychological impairment in 34.2% of HIV-positive subjects, and in 39.7% of HIV-positive subjects coinfected with the hepatitis C virus [11]. The authors concluded that the prevalence, severity and pattern of cognitive impairment were similar to those reported by western studies. A preliminary study addressing the effect of HAART on neurological function in Uganda found that HIV dementia improved from 61% at baseline to only 4% after six months of HAART [12].

Cognitive impairment and depression frequently coexist in HIV [13]. Despite this, few neuropsychological studies of HIV-positive individuals in developing countries have included adequate measures of depression. Depression can adversely influence performance on cognitive tests due to poor effort, slowed processing speed, psychomotor retardation, or a combination of these factors [14]. The IHDS may be particularly vulnerable to the effects of depression since two of the three subtests have time limits designed to measure speed of information processing and motor speed.

To address these questions, this preliminary study focused on three objectives. The first was to explore the use of the IHDS in HIV-positive individuals in Botswana to measure the prevalence of neurocognitive impairment. Recent guidelines [15] support the use of standardized mental status examinations, such as the IHDS [16], to diagnose HAND in regions where neuropsychological testing is not available. The second objective was to compare performance on the IHDS with performance on the most sensitive test combination for detecting HAND, verbal learning/memory and processing speed [17]. The third was to explore the association between performance on the IHDS and important variables, such as depression, age, education and current CD4 count.

## Methods

Participants were 120 randomly selected HIV-positive subjects (60 women and 60 men). They were asked to participate in this study during routine follow-up visits from March to May 2008 at the Infectious Disease Care Clinic at Princess Marina Hospital, a 550-bed tertiary referral hospital in Gaborone, which serves southern Botswana. Control subjects were not included because this pilot study was focused on determining the feasibility of adapting and validating neuropsychological tests and depression inventories used in developed countries for HIV-infected individuals in Botswana. No reimbursement for participation was offered.

Inclusion criteria were: (1) documented HIV-positive status; (2) age of 21-50; (3) ambulatory status; (4) the ability to comprehend study procedures; and (5) provision of informed consent. Consistent with current treatment practices in Botswana at the time of this study, subjects started treatment with HAART when their CD4 cell count dropped to 200/mm^3 ^or below. Thus, most patients in this study had a history of very low CD4 counts since 97.5% were prescribed HAART.

Exclusion criteria eliminated individuals with cognitive impairment unrelated to HIV, such as: (1) neurological conditions (e.g., head injury, seizure disorder); (2) medical illness unrelated to HIV (e.g., chronic hepatic or renal failure, malignancy) or severe HIV-related disease (current opportunistic infection); (3) current fever; (4) severe psychiatric disorder (e.g., schizophrenia); (5) a history of substance abuse; and (6) inability to function independently.

### Procedure

Participants underwent a standardized neuropsychological examination and assessment of depression, activities of daily functioning, and subjective cognitive complaints. Details of the depression findings are reported elsewhere [18]. Assessments were carried out by Botswana nursing staff and neuropsychology researchers from the University of Pennsylvania. Testing procedures were standardized across examiners; the neuropsychology expert observed all examiners in training prior to test administration to subjects.

Structured interviews and chart reviews were performed to obtain information about medical and psychiatric history, pattern of substance use, marital status, education, current medications, recent CD4 counts and viral loads. Viral load testing was performed using the Amplicor HIV-1 Monitor Test (Roche Molecular Systems, Branchburg, New Jersey). Viral load measurements have been categorized dichotomously (detectable vs. undetectable) using a threshold of 400 copies/ml. The investigators evaluated neuropathy using a targeted history composed of structured validated questions designed to elicit symptoms of distal sensory loss or pain.

This research study was approved by the Institutional Review Boards of the Botswana Ministry of Health, Princess Marina Hospital, and the University of Pennsylvania. All consent forms, questionnaires, and tests were translated into Setswana and back translated. For patients with limited reading ability, the examiners orally administered the Activities of Daily Living (ADL) Scale.

### Measures

The test battery was selected to assess multiple cognitive-motor ability domains that have repeatedly been shown to be affected by HIV-associated brain disease in both developed and developing countries.

#### International HIV Dementia Scale

The IHDS is a screening measure of neurocognitive impairment that includes: memory registration for four common objects; motor speed involving the rapid tapping of the thumb and first digit of the non-dominant hand; speed of information processing and/or executive functioning measured by repetition of a three-position alternating hand sequence; and memory recall of the four objects [16]. A prior study in Uganda determined the optimal cut-off value for the IHDS. The cut-off value of 9.5 maximized the sensitivity (71%) and specificity (79%) for HIV dementia, and thus was chosen for our current study. This cut-off score has been recommended to minimize false positives errors [16].

#### World Health Organization-University of California Auditory Verbal Learning Test

The World Health Organization-University of California Auditory Verbal Learning Test (AVLT) is a list-learning task that assesses verbal learning and memory with 15 words carefully selected to be familiar in most cultures [19]. Scores include the total number of words learned for all five trials (TL), delayed recall (DR), and recognition of the words (R).

#### Wechsler Adult Intelligence Scale (third edition) Digit Symbol Coding

The Digit Symbol (DS) Coding is a paper-pencil measure of processing speed in which subjects use a key of digit-symbol pairs at the top of the test page and are required to fill in the correct symbol for each number as quickly as possible within a 120-second time limit. The score is the number of correct items completed within the time limit [20].

#### Mood Module of the primary care evaluation of mental disorders

The Mood Module (MM) [21] is a focused interviewing guide that follows the Diagnostic and Statistical Manual-IV (DSM-IV) criteria [22] for screening medical patients for current depression. It is composed of simple "yes" and "no" questions. If five or more of the nine symptoms are present and one of these symptoms is sadness/hopelessness or anhedonia, then a diagnosis of major depressive disorder (MDD) is supported. The MM has been successfully used to diagnose depression in patients with HIV and was found to have a sensitivity of 77% and specificity of 84% [23].

#### Activities of Daily Living Scale

This questionnaire was adapted from the original measure and selected for its wide use and demonstrated validity in studies of medically ill and dementia populations, including HIV [24]. It is a 14-item scale measuring physical self-maintenance activities (e.g., dressing, bathing) and instrumental activities of daily living (e.g., preparing meals, taking medications). Each item is rated on a four-point scale: (1) no difficulty at all; (2) has some difficulty; (3) needs some assistance; or (4) can't do at all. Thus, higher scores indicate more impairment in daily functioning. The modified version used in this study was used and validated in studies of HIV subjects [25].

#### Subjective Cognitive Complaints questionnaire

This is a brief self-report questionnaire of subjective cognitive complaints with specific items for four cognitive domains: memory, concentration, speech and thinking. This simple questionnaire asked patients to rate the amount of difficulty they were experiencing on a scale of 0 to 2, as follows: 0 no problem; 1 yes, but currently absent; 2, yes, currently present. Scores for each cognitive domain were added together for a total subjective cognitive score, with a maximum possible score of 8. This questionnaire was previously used in a cross-cultural study that assessed cognitive complaints in HIV-positive individuals in five different countries [4].

### Data analysis

We performed descriptive analyses and comparisons to examine the demographic and clinical characteristics of patients. All analyses were conducted using StataMP 10.0 (Stata Corp., College Station, Texas). Correlation coefficients were used to assess relationships between continuous variables. Student's t-tests and analysis of variance were used for comparisons of normally distributed variables. Kruskal-Wallis rank was used for non-normal variables, and Fisher's exact tests were used for categorical variables. The relative importance of dementia on cognitive outcomes, adjusted for demographic (age, race, gender, education, language of test administration, depression), and HIV (current CD4 count, time since beginning HAART, a detectable viral load) characteristics, was assessed using regression models. As this was an exploratory study, alpha was set at 0.05 to determine statistical significance.

## Results

Demographic and clinical characteristics of the cohort are described in Table 1.

**Table 1:** Demographic and clinical characteristics of subjects (n = 120).

	PERCENT	MEAN	STANDARD DEVIATION	RANGE
**CHARACTERISTICS** HIV-POSITIVE SUBJECTS (n = 120)				

Age (years)		37.5	6.5	23 - 50

Education (years)		8.9	4.1	0 - 18
No education	8%			
Primary (1-7 yrs)	29%			
Secondary (8-12 yrs)	48%			
Post-secondary (>12 yrs)	15%			

Gender (female)	50%			

CD4 count		360.4	181.4	42 - 881.8
Subjects with CD4 <200/mm^3^	20%	149.2	44.2	42 - 199.4

Viral load				
<400 copies/mL	80%			
>400 copies/mL	17%	37,181.0	92,828.2	490 - 270,000
Unknown	3%			

Time since HIV-positive diagnosis (years)		3.9	2.4	<1 - 14

On HAART (≥ 3 drugs)	97.5%			

Time since beginning HAART (years)		2.8	2.0	<1 - 7

Marital status:				
Single	75%			
Married	23%			
Widowed	2%			

Employment status:				
Employed	68%			
Unemployed	32%			

Language of test Administration:				
Setswana	39%			
English	52%			
Both	9%			

### International HIV Dementia Scale

Using a cut-off score of 9.5 or less on the IHDS, 38% of the patients met criteria for neurocognitive impairment (Table 2). Subjects diagnosed with neurocognitive impairment on the IHDS had lower scores on the AVLT for both TL (p = 0.023) and DR (p = 0.026), but not for R (p = 0.897). Similarly, those classified with neurocognitive impairment on the IHDS had slower processing speed on the Digital Symbol (DS) coding (p < 0.001).

**Table 2:** Mean scores and standard deviation (in parentheses) for the two cognitive tests, Auditory Verbal Learning (AVL) Test and Total Learning (TL) score, and Digit Symbol (DS) Coding (number of items correctly completed within the time limit), for subject groups classified with and without neurocognitive impairment (IHDS cut-off score of ≤ 9.5).

	SUBJECTS NOT IMPAIRED WITH IHDS (62%)	SUBJECTS IMPAIRED WITH IHDS (38%)	P-VALUE
**NEUROPSYCHOLOGICAL MEASURES**			

WHO Auditory Verbal Learning Test (AVLT) (Total Learning, TL)	47.61 (9.11)	43.78 (8.42)	p = 0.023

Digit Symbol Coding (DS) (items completed correctly within time limit)	41.79 (14.47)	30.40 (11.98)	p < 0.001

Current CD4 count (p > 0.445), gender (p = 0.556), substance use (p = 0.194) and employment status (p = 0.419) were not associated with neurocognitive impairment. Language of test administration was not associated with performance on the IHDS (p = 0.613). There was no association between peripheral neuropathy and neurocognitive impairment (p = 0.389). In addition, there was no association between neurocognitive impairment and length of time since HIV diagnosis (p = 0.528). However, subjects with neurocognitive impairment had been prescribed HAART for longer than those without impairment (p = 0.026).

Subjects classified with neurocognitive impairment were characterized by increased age (p = 0.008) and had fewer years of education (p = 0.035). Looking at the three subtests of the IHDS, increased age (p = 0.039) and those with less years of education (p = 0.002) had slower motor speed (finger tapping). Similarly, information processing speed (serial hand positions, p = 0.005) was adversely affected by increasing age (as subjects became older, they were slower), but level of education did not affect performance on this subtest (p = 0.195). Performance on the memory subtest was not associated with age (p = 0.381) or education (p = 0.778). Because of the effect of education, we re-analyzed the IHDS data excluding subjects with no education. With this adjustment, 35% of subjects met criteria for neurocognitive impairment.

MDD was diagnosed with the MM in 24% of subjects, but depression was not significantly associated with the total score on the IHDS (p = 0.620) or scores on any of the subtests of the IHDS (motor speed p = 0.448, psychomotor speed p = 0.973, memory p = 0.803).

### Auditory Verbal Learning Test

Age was not significantly associated with verbal leaning/memory with the AVLT (TL p = 0.460, DR p = 0.841, R p = 0.245). Similarly, gender (TL p = 0.291, DR p = 0.533, R p = 0.340), substance use (TL p = 0.084, DR p = 0.678, R p = 0.555), employment status (TL p = 0.786, DR p = 0.469, R p = 0.149), CD4 count (TL p = 0.747, DR p = 0.674, R p = 0.165), time since beginning HAART (TL p = 0.313, DR p = 0.81, R p = 0.395), and depression (TL p = 0.604, DR p = 0.852, R p = 0.553) were not associated with verbal learning/memory.

Level of education was not associated with total learning (TL) on the AVLT (p = 0.758), but was significant for delayed recall (DR) (p = 0.027) and did not quite reach statistical significance for recognition (R) (p = 0.053). Language of test administration was not associated with AVLT TL (p = 0.065), but those tested in Setswana had higher DR scores (p < 0.001), and subjects tested with a combination of both languages had lower R scores than those tested in either English or Setswana alone (R p = 0.009).

In fully adjusted analyses (all demographic and HIV characteristics) for AVLT TL, neurocognitive impairment did not reach statistical significance (b = -3.79, p = 0.064). However, due to the limited sample size in this pilot study, we also reduced the final model down to only significant effects. In this final model, neurocognitive impairment and language of test administration were significantly associated with AVTL TL. Subjects with neurocognitive impairment tended to have four-point lower TL scores (b = -4.13, p = 0.013), while subjects tested in English had four-point lower TL scores than subjects tested in Setswana (b = -4.35, p = 0.011).

In fully adjusted models for AVLT DR, both neurocognitive impairment and language of test administration still significantly impacted the scores. Subjects with neurocognitive impairment tended to have 1.5-point lower DR scores (b = -1.48, p = 0.015), while subjects tested in English had two-point lower DR scores than subjects tested in Setswana (b = -1.64, p = 0.032).

### Digit Symbol Coding subtest

There was an inverse relationship between a subject's age and processing speed (Digital Symbol, DS); as the subjects' ages increased, they performed significantly slower than their younger counterparts (p < 0.001). Similarly, subjects with less years of education were also slower (p < 0.001); for each additional year of education, subjects completed an average of 2.3 additional items. Males were significantly slower than females (p = 0.032), and subjects tested in Setswana were slower than subjects tested in either English (p < 0.001) or both English and Setswana (p < 0.001).

Processing speed was not affected by substance use (p = 0.194), employment status (p = 0.470), CD4 count (p = 0.197), or time since beginning HAART (p = 0.656). In addition, there was no observed association between processing speed and depression (p = 0.345). Twenty-six percent of subjects reported symptoms of peripheral neuropathy, but there was no association between those with and without these symptoms for processing speed on the DS (p = 0.865).

In fully adjusted models, neurocognitive impairment and education were the only characteristics associated with DS. Subjects with fewer years of education were slower, with a decrease of two points for each year of missed education (b = 1.76, p < 0.001). Subjects with neurocognitive impairment had DS scores that were seven points lower on average than subjects without neurocognitive impairment (b = -6.80, p = 0.004).

### Activities of Daily Living Scale and Subjective Cognitive Complaints questionnaire

Individuals who met criteria for a MDD on the MM reported greater levels of impairment (p = 0.044) on the ADL Scale, but classification of neurocognitive impairment with the IHDS was not related to ADL scores (p = 0.334). Similarly, speed of information processing (DS p = 0.395) and learning/memory (TL p = 0.082, DR p = 0.275, R p = 0.188) were not associated with ADL scores. There was no association between the ADL score and CD4 level (p = 0.949), age (p = 0.482) or education (p = 0.081). Similarly, there was no significant association between neurocognitive impairment and subjective cognitive complaints (total score, p = 0.920).

### Viral loads

Eighty percent of subjects in our sample did not have a detectable viral load (i.e., below the detection limit of 400 copies/mL). Thus, it was not feasible to analyze the data as a continuous outcome. We did look at viral load as a dichotomous outcome of yes/no detectable viral load. However, there were no significant associations with this variable, even at a bivariate level.

## Discussion

The prevalence of neurocognitive impairment in our study was higher than anticipated. Most of our subjects were receiving treatment with HAART, thus we did not expect so many to meet criteria for neurocognitive impairment on a screening measure, such as the IHDS. Yet it is important to note that the high prevalence of neurocognitive impairment in this study may reflect our use of a tool, such as the IHDS, which requires further refinement. Given the association between demographic variables and performance on the IHDS, future studies in Botswana should establish demographically adjusted normative standards for the IHDS, as has been done for a similar screening measure, the HIV Dementia Scale, in the United States [26].

A study in Uganda using the IHDS found HIV dementia in 31% of subjects, but in that study, less subjects were receiving antiretroviral treatment [16]. Furthermore, another recent study in Uganda demonstrated significant improvement in cognitive functioning after the initiation of HAART [12]. Different viral clades may account for the variation in the incidence of dementia in geographic distinct regions of Africa. Certain clades may be more or less neuropathogenic [27,28].

For example, in Ethiopia, the IHDS did not detect impairment in untreated HIV-positive subjects, and Clifford *et al *hypothesized that clade C virus, which is most prevalent in Ethiopia, may be less neurotropic than clades A and D, which are predominant in Uganda [8]. However, the researchers in Ethiopia modified the IHDS memory sub-test stimuli, which may have affected the sensitivity of the test. In addition, subjects in the Ethiopian study were recruited from the community, rather than from an HIV clinic, and therefore may have been healthier than the subjects in the Uganda study and those in our study.

Furthermore, the relatively high prevalence of neurocognitive impairment in HIV-positive subjects found in our Botswana study, a region where clade C HIV predominates, is consistent with several Pacific Rim studies. Authors of one Pacific Rim study concluded that that clade C HIV is no less neurotropic than other clades [9]. In addition, a neuropsychological study in southern India of clade C-infected HIV-positive subjects found mild to moderate cognitive impairment in 60% of subjects who did not have any clinically identifiable functional impairment [10].

Neurocognitive impairment and depression are independent complications of HIV. For example, although 24% of our subjects were diagnosed with MDD, depression did not affect the total score or any of the three subtest scores of the IHDS. Similarly, depression was not significantly associated with verbal learning/memory (the AVLT) or processing speed (DS Coding), which is consistent with prior studies showing that depression and cognitive impairment are independent in HIV [29,30]. Furthermore, subjects classified with neurocognitive impairment using the IHDS were basically asymptomatic since they did not report problems with daily activities or complain of cognitive problems. This was consistent with the examiners' observations that the majority of patients were independent, with minimal functional limitations or complaints. In addition, 68% of subjects reported being gainfully employed.

The results of our study may have implications for when it is best to start HIV therapy. The number of years since HIV diagnosis was not a significant predictor for neurocognitive impairment. However, the longer a subject was prescribed HAART, the more likely he or she was to be diagnosed with neurocognitive impairment. Persistent cognitive deficits in HAART-treated subjects may be a result of pre-treatment neurological damage, poor central nervous system penetration of some antiretroviral agents, drug resistance, poor adherence, potential neurotoxicity, and co-morbidities [31]; future prospective studies in Botswana will need to clarify these issues.

Continuous viral loads should generally be log-transformed in order to meet normal assumptions, but in our sample, 80% did not have a detectable viral load (i.e., below the detection limit). Thus, it was not feasible to analyze the data as a continuous outcome. We did look at viral load as a dichotomous outcome of yes/no detectable viral load, but there were no significant associations with this variable even at a bivariate level.

The finding that current CD4 count was not associated with performance on the IHDS or the other two cognitive tests was expected, since most of the subjects were receiving HAART. Studies in the HAART era have shown that biomarkers, such as current CD4 count, plasma viral load, CSF viral load and CSF immune markers, are not correlated with, or predictive of, neurocognitive impairment [32]. Recent research has suggested that cognitive impairment may become more likely the lower the value of the nadir CD4 cell count [11,33,34]. Nadir CD4 cell count was not available for our subjects, but should be included in future research in Botswana.

The IHDS has the potential to be a useful screening instrument for HIV-associated neurocognitive disorders (HAND). As anticipated, subjects who were classified with neurocognitive impairment on the IHDS also performed significantly lower on other tests of cognitive function, specifically processing speed (DS Coding) and verbal learning/memory (the AVLT). On a cautionary note, it is sobering that several variables also affected performance on the IHDS and the two other cognitive measures. For example, language of test administration was related to performance on tests of both verbal memory (AVLT) and processing speed (DS), which may be related to cultural factors or socio-economic status, and requires further investigation.

Even though there was a relatively small range of ages in our sample, age adversely affected performance on the IHDS. Similarly, education was associated with performance on the IHDS. Older and less educated subjects obtained significantly lower scores on the IHDS. Performance on most mental status examinations does not depend on processing or motor speed, but speed is a major component of two of the three IHDS subtests; thus performance is particularly vulnerable to the effects of age and education. This highlights the need to take these factors into account when determining local normative cut-off scores for screening tests of HIV dementia [26].

Our results support the hypothesis that memory impairment in HIV infection is caused by disruption of subcortical processes. This is reflected in impaired free recall due to retrieval inefficiencies, but relatively preserved recognition. Subjects meeting criteria for neurocognitive impairment on the IHDS performed poorly on the learning/memory test (AVLT) for both total learning (TL) and delayed free recall (DR), but not recognition (R). This is consistent with the neuropsychological pattern of memory deficits documented in prior studies of HAND, characterized by impaired learning and retrieval, but intact recognition [35-37].

This pilot study has several limitations: the absence of an HIV-negative control group for comparison; symptoms of peripheral neuropathy being elicited by a targeted clinical history and not by examination; and the study being conducted among HIV-positive patients in an urban outpatient HIV clinic in Botswana, which may not be representative of HIV-positive individuals in community and rural settings. In addition, appropriate demographically corrected norms and determination of possible learning effects with repeated screenings are needed for the IHDS before it can be used confidently to classify HAND in individuals in Botswana.

## Conclusions

More than one-third of HIV-positive subjects met criteria for neurocognitive impairment with the IHDS, despite the fact that almost all were treated with HAART for an average of two years and were asymptomatic from the perspective of subjective complaints and everyday activities. This finding, combined with recent research indicating that cognitive impairment may become more likely the lower the value of the nadir CD4 cell count, gives further weight to the importance of initiating treatment with HAART at earlier stages of the disease process.

In addition, there may be more than one explanation for the persistence of neurocognitive deficits, with different policy implications. These include: treatment regimen, particularly poor central nervous system penetration of some antiretrovirals; drug resistance; poor adherence; potential neurotoxicity; and co-morbidities, such as long-term cardiovascular disease side effects of HAART, and chronic HIV brain infection. Our results, while admittedly tentative, indicate that neurocognitive impairment is likely to be an important component of HIV infection in developing countries, and highlight the need to develop effective treatments.

## Competing interests

The authors declare that they have no competing interests.

## Authors' contributions

KL conceived the study design, supervised and participated in the data collection and psychological evaluation of subjects, participated in the statistical analysis, and drafted the manuscript. AS participated in the study design, data collection, and revisions of the manuscript. ES contributed to the study design and revisions of the manuscript. MM participated in the study design, provided supervision for the overall collection of data in the Infectious Disease Care Clinic, and undertook revisions of the manuscript. SR participated in the study design and provided statistical analysis and interpretation of data. KS participated in the data collection, psychological evaluation of subjects, and revisions of the manuscript. RN participated in the data collection and psychological evaluation of subjects. All authors read and approved the final manuscript.
